# Abstract Words as Social Tools: Which Necessary Evidence?

**DOI:** 10.3389/fpsyg.2020.613026

**Published:** 2021-01-15

**Authors:** Anna M. Borghi, Claudia Mazzuca, Federico Da Rold, Ilenia Falcinelli, Chiara Fini, Arthur-Henri Michalland, Luca Tummolini

**Affiliations:** ^1^Department of Dynamic and Clinical Psychology, and Health Studies, Sapienza University of Rome, Rome, Italy; ^2^Institute of Cognitive Sciences and Technologies, Italian National Research Council, Rome, Italy; ^3^Department of Psychology, University of York, York, United Kingdom; ^4^University of Montpellier—LIFAM, Montpellier, France

**Keywords:** abstract concepts, deaf children, autism, sociality, gender, interoception, grounded cognition, embodied cognition

## Introduction

Recent theories on abstract concepts and words (ACs), such as Words As social Tools (WAT) (Borghi et al., 2019b) and Language is an Embodied Neuroenhancement and Scaffold (LENS) (Dove, 2019) have underlined the crucial role of both sensorimotor experience and language for ACs representation and use [see Dove et al. (2020), for a comparison]. Here we focus on the WAT view. WAT highlights the role of language, sociality, and inner grounding (interoception, metacognition) for ACs. Furthermore, WAT seeks to integrate a developmental perspective with approaches focusing on conceptual use and brain representation. We briefly illustrate evidence coming from both clinical and non-clinical populations and identify areas where additional evidence that ACs evoke linguistic, social, and interoceptive experience is still needed.

## Linguistic Experience

WAT posits that language is paramount for ACs. Linguistic explanations help us form categories composed of perceptually different exemplars. Importantly, according to WAT not only outer but also inner speech plays a significant role for ACs. Because we are less confident about the meaning of ACs, we would rely more on inner speech in their use than with concrete concepts (CCs). WAT proposes that we use metacognition to monitor our knowledge (Shea, 2018). This monitoring process leads to two different outcomes, both of which involve inner speech (Borghi et al., 2020): we would continue to inner search for meaning and/or we would prepare ourselves to seek information from others [see the notion of social metacognition in Borghi et al. (2019a)]. In support of this view, evidence on the processing of ACs in non-clinical samples of children and adults suggests an active engagement of the mouth articulatory system, which might be the physiological correlate of inner speech [for very recent evidence see Barca et al. (2020) and Villani et al. (2020); review in Borghi et al. (2019a)]. This evidence points to the importance of the embodied experience of language for both the acquisition (the activation of the mouth motor system would also express the re-enactment of the conceptual acquisition experience) and consolidation in memory of ACs.

### DHH Children

Understanding how Deaf and Hard of Hearing (DHH) children engage with ACs might offer useful insights on their acquisition. Indeed, DHH children generally underperform other children in spoken language and language comprehension. According to the Language Scaffolding hypothesis, DHH difficulties are not due to auditory problems but to linguistic deprivation. As a recent article states in its title, “Deaf children need language, not (just) speech.” (Hall et al., 2019): only 1–2% of deaf children worldwide receive a sign language education (Haualand and Allen, 2009). Evidence consistent with this hypothesis indicates that deaf children with deaf parents, exposed to sign language before cochlear implantation, do not have executive function deficits and that they outperform non-signing children in linguistic tasks and perform similarly to hearing ASL-English bilinguals with deaf parents (e.g., Davidson et al., 2014; Marshall et al., 2015).

What about ACs? Considering the social context in which we employ language (Fini and Borghi, 2019), WAT predicts that children who do not share a language (signed or spoken) with their parents have more difficulties with the acquisition of ACs than children with signing parents. Moreover, if acoustic rather than signed or written input is essential for conceptual acquisition, deaf children who use sign language should be less competent with ACs. This may be true only for acoustically salient terms, as onomatopoeia (“ouch”). Finally, inner speech used by deaf signers (Lœvenbruck et al., 2018) consists of internal representations of signs rather than phonological representations (Perrone-Bertolotti et al., 2014). Both speech and signs can activate inner language production, and they appear to have similar neural underpinnings. How does this impact ACs?

Unluckily, evidence on ACs in DHHs is scarce. To our knowledge, two sources of evidence are available. The first comes from studies building on the hypothesis that deaf children with hearing parents have deficits in Theory of Mind (ToM) (Peterson and Siegal, 1995). ACs are acquired thanks to verbal interactions, which also reinforce the ToM development. For example, there is a significant relationship between mothers' conversation about mental state concepts and the development of ToM in 4-6-years-olds (Moeller and Schick, 2006).

The second source of evidence consists of systematic studies that revealed an enhanced difficulty of DHHs in comprehending linguistically acquired concepts, like ACs. Such difficulty, not present in 6-years-olds, develops progressively with age. In most cases, children had both hearing parents, and deafness onset was before three. Wauters et al. (2006) tested reading comprehension and word identification of more than 400 Dutch deaf students (age 6–20). Deaf children lag behind the others in reading comprehension but did not differ from them in identifying words. To explain this difficulty, the authors refer to the progressively higher number of terms acquired *via* linguistic explanations rather than perceptually. In a further study, Wauters et al. (2008) compared deaf and hearing children between 7 and 15 years of age on a self-paced reading task and comprehension of sentences. Their results highlight both the importance of Modality of Acquisition (MoA) and a difference between deaf and hearing participants; they also indicate that deaf children are impaired not only with linguistically acquired words, but also with perceptually acquired ones (e.g., “The boy smelled *soup* / *gas* in the kitchen”). Significantly for us, there was an effect of instructional age on reading times limited to hearing participants, likely due to their improvement with linguistically acquired words. Though compelling, these results are limited by the fact that they do not report the proficiency of participants in sign language.

Summarizing, the linguistic performance of DHH children has some limitations. However, further evidence is needed to investigate difficulties with ACs. In the studies we reviewed, the majority of children have hearing parents, hence their difficulties are more likely to occur due to the absence of language than of acoustic information. Further studies are needed, in which deafness of parents and mastery of sign language are controlled (Scorolli, 2019). Further research is also necessary to investigate the role of mouth articulation, which we hypothesize to be strictly related to inner speech in non-signers. It would be important to understand whether this mechanism is less prominent in sign speakers. Articulated inner speech may be substituted by signed inner speech.

### Elderly and Semantic Dementia

Healthy elderly is an interesting population to test eventual decline in ACs use, primarily because their linguistic knowledge is notably well-preserved. The literature on Age of Acquisition would lead to predict a selective decline of ACs. However, such a drop might be limited because linguistic networks, preserved in the elderly, are more relevant for ACs than CCs [for a review, see Borghi and Setti (2017)]. Evidence consistent with this hypothesis shows that the elderly rely on phonological/linguistic and visual features more than younger adults (e.g., Roxbury et al., 2016). These activations might compensate for the loss of the later acquired ACs. Unfortunately, however, evidence on ACs in the elderly is scarce and sometimes conflicting.

Similarly, contrasting evidence on semantic dementia has generated heated debates. While various studies identified a reversal of the concreteness effect (i.e., patients preserved their competence with ACs better than with CCs; e.g., Bonner et al., 2009), recent work reported an opposite decline of ACs [e.g., (Hoffman and Lambon Ralph, 2011); short review in Dove (2019)]. Even though the controversy is beyond the scope of this paper, the presence of reversed concreteness effects is very relevant because these effects are clearly associated with linguistic knowledge and use. Evidence of semantic dementia is crucial also because it is linked to lesions in the anterior temporal lobe (ATL). ATL is an area deemed critical to ACs: hybrid models (i.e., models integrating the view that concepts are grounded with the view that meaning is defined in terms of lexical co-occurrences), posit that modal, and amodal information converges in ATL (Pobric et al., 2009; Hoffman et al., 2018). Given the conflicting evidence on semantic dementia and ACs, further in-depth studies are needed.

## Social Experience

According to WAT another crucial aspect for ACs is sociality. Other people help us in forming categories whose members are not perceptually similar; furthermore, we typically rely on other people to understand ACs' meaning. If sociality is crucial for ACs formation and inner speech for their processing, then populations who experience limitations in social interaction should experience difficulties in mastering abstractness.

Individuals with ASC (Autism Spectrum Condition) and people diagnosed with schizophrenia–particularly patients who experience auditory verbal hallucinations (sz-AVH)–share some social behaviors: they have communication difficulties and tend to avoid eye contact (Dvir et al., 2011). Furthermore, their use of inner speech is anomalous: while individuals with ASC generally avoid using inner speech, the inner speech of individuals with sz-AVG is intruded by negative thoughts and auditory hallucinations (Petrolini et al., 2020).

Classical descriptions associated schizophrenia with concretism (i.e., the tendency to interpret language literally). Recent evidence indicates that schizophrenic patients perform worse than controls across different kinds of figurative expressions–e.g., metaphors, idioms, and proverbs–and with different tasks (multiple choice, verbal explanations) [see also Mossaheb et al. (2014), Parola et al. (2018), Bambini et al. (2020)]. While this evidence looks promising for supporting the WAT perspective, further studies are needed to investigate selective difficulties with abstract concepts–and not only with metaphorical expressions–and to assess whether a selective deficit in the processing of abstract concepts, rather than a more general cognitive impairment, accounts for the worse performance concerning the figurative domain.

Evidence on autism and abstractness is scattered [for a concise review, see Borghi et al. (2019b)]. Children with ASC do not differ from controls in the categorization of basic and superordinate concepts. However, they need to visualize items and situations, have difficulties with mental states terms, and rarely make use of inner speech (Petrolini et al., 2020). Hence, their mastering of ACs–except for numerical concepts (e.g., Iuculano et al., 2014) which are considered as less abstract than other abstract concepts such as e.g., philosophical ones–could be limited, even if this might be less true for individuals with high-functioning autism (including Asperger individuals). Bayesian approaches have interpreted autism as a difficulty to activate inferences on social actions, owing to the scarce capability in monitoring the discrepancy between their own and others' mental states (Palmer et al., 2017; Deschrijver and Palmer, 2020). Some studies link autism with auditory problems [(Siegal and Blades, 2003); review in O'connor (2012)], more pronounced with speech stimuli, and ascribe to them the subsequent difficulty to engage in joint attention and action. Further studies are thus needed to disentangle the role that sociality and auditory efficiency play for ACs in ASC.

## Embodied and Sociocultural Experience

While in the previous section we focused on the role of social interaction, here we consider more broadly the experience of being embedded in a sociocultural environment—with its norms and traditions. According to WAT, both embodied and sociocultural factors influence ACs, which vary more than concrete ones across populations, cultures, and languages.

Consider gender. Current understandings propose that both abstract, sociocultural factors (e.g., social norms, cultural environment) and concrete, embodied, and biological ones (e.g., biological asset) are relevant in shaping gendered identities [(Fausto-Sterling, 2019); for a review see Hyde et al. (2019)]. However, these components are differently salient, depending on specific experiences and cultures (Mazzuca et al., 2020b). Mazzuca et al. (2020a) compared the features produced in a free-listing task in association to the word “gender” by Italian cis-gender, monosexual participants (normative) and gender-diverse, non-monosexual ones (non-normative). While normative individuals mainly conceptualized gender in concrete terms–e.g., they frequently mentioned words related to the biological and perceptual sphere–, non-normative participants consistently relied on more abstract words—e.g., *queer, fluidity, construction*. Similarly, English-speaking cis-gender individuals mentioned more biological and less sociocultural content in their definitions of woman/man and female/male than transgender individuals (Schudson et al., 2019). In addition, gender/sex minority groups also gave more complex definitions of *feminine, masculine, female*, and *male* than gender/sex majority groups. These findings suggest that language and sociality shape gender-conceptualizations, along with embodied factors. People whose experiences with gender differ from bigender, heteronormative standards (e.g., gender-diverse; transgender; plurisexual individuals) more likely rely on abstract information in their representation of gender-related concepts. This representation, while being mediated by bodily experiences, also relies on sociocultural understandings of gender, conveyed through language, and might involve ACs to a greater extent. Investigating the conceptualization of gender through more tailored cross-cultural/cross-linguistic studies, and using an intersectional approach is therefore one of the possible future directions in the study of ACs—as it might shed light on the relative contribution of both sociocultural and experiential components of abstract conceptual knowledge.

## Interoceptive Experience

According to WAT, interoceptive information (bodily signals on inner states) such as heart beating– plays a critical role in ACs, particularly emotional ones [see also Connell et al. (2018); evidence in Villani et al. (2020)]. Data relevant to WAT pertains to populations whose interoceptive sensitivity changes in particular phases of their life, or due to specific identity and bodily experiences.

We will outline two examples. Consider pregnant women. Due to their specific condition, they might be particularly sensitive to interoceptive information as confirmed by preliminary experimental evidence (Porciello et al., 2019). This enhanced sensitivity might result in a different way to represent ACs, for which interoceptive experience is critical. Preliminary data in our lab support this hypothesis. When asked to evaluate how much target words evoke interoception, ratings of pregnant women are significantly higher than those of controls. Importantly, this effect is present with ACs, and not with CCs. A similar enhanced interoceptive sensitivity leading to a different representation of ACs might characterize transgender individuals, who might have experienced significant inner and outer bodily transformation. Although preliminary, these results advocate for further research exploring the relationship between interoception and different inner-bodily experiences in modulating abstract conceptual knowledge.

## Conclusion

Different kinds of evidence can be critical to test the main tenets of WAT. Among these, here we suggested that neurodevelopmental, neuropsychological, psychiatric evidence, and research on specific populations in particular phases of their life (e.g., pregnant women) or with different identity experiences (e.g., people with non-conforming gender identities) might be pursued to assay some of the WAT predictions on ACs. More generally, this evidence can contribute to test proposals according to which embodiment, language, and sociality are crucial for ACs representation and use. Further research is needed to address some of the raised questions and to formulate new ones.

## Author Contributions

AB and CM wrote the manuscript. FD, IF, CF, A-HM, and LT provided revisions and comments. All the authors conceived the manuscript.

## Conflict of Interest

The authors declare that the research was conducted in the absence of any commercial or financial relationships that could be construed as a potential conflict of interest.
